# The distribution characteristics of PD-1 pathway-related immune cells in esophageal cancer tissue and their prognostic significance

**DOI:** 10.1371/journal.pone.0325349

**Published:** 2025-06-30

**Authors:** Dehua Kong, Chunyan Gao, Yang Yu, Lu Yang, Ji Ma, Shimin Tang, Ying Mao, Yong Li, Na Li

**Affiliations:** 1 Department of Oncology, Suining Central Hospital, Suining City, China; 2 Department of Oncology and Hematology, Shenzhen Pingshan District Central Hospital, Shenzhen City, China; 3 Department of Radiological Imaging, Suining Central Hospital, Suining City, China.; University of Tennessee Health Science Center, UNITED STATES OF AMERICA

## Abstract

**Objective:**

This study aims to elucidate the distribution patterns of immune cells associated with the programmed cell death protein 1 (PD-1) pathway within esophageal cancer (EC) tissues and to determine their correlation with patient prognosis.

**Methods:**

We included tissue samples from 236 EC patients who had undergone surgery at our institution between January 2016 and January 2021. This study examined the correlation between six immunohistochemical markers and the clinical profiles of these patients. Survival analysis was performed using the Kaplan-Meier method and the LOG-rank test to evaluate the impact of immunohistochemical marker expression on patient survival. A clinical predictive model was developed and validated for prognostic assessment.

**Results:**

Expression levels of PD-1, PD-L1, FOXP3, and CD25 were found to be positively associated with the depth of tumor invasion and lymph node metastasis (P < 0.05). In contrast, CD4 and CD8 expression levels were inversely related to these parameters (P < 0.05). High expression of PD-1, PD-L1, FOXP3, and CD25, along with lymph node metastasis, were identified as independent prognostic risk factors (P < 0.05). Patients with low expression of PD-1, PD-L1, FOXP3, CD25, and high expression of CD4 and CD8 exhibited improved three-year survival rates (P < 0.001). The predictive model, based on these factors, demonstrated high discrimination and accuracy.

**Conclusion:**

A prognostic model incorporating the expression levels of PD-1, PD-L1, FOXP3, CD25, and lymphocyte infiltration offers robust predictive validity for the prognosis of EC patients.

## Introduction

Esophageal cancer (EC) is a malignant epithelial tumor of the esophagus that poses a serious threat to patient survival [1–3]. Early symptoms are often subtle, leading to a majority of patients being diagnosed at an advanced stage, which contributes to a poor survival rate [4]. According to the global cancer statistics for 2022, EC has an incidence rate of 510,716 cases, making it the 11th most common malignancy. Furthermore, with 445,129 deaths, it is the 7th leading cause of cancer-related mortality [1]. In China, EC is particularly prevalent, with 95.5% of cases being squamous cell carcinoma, predominantly located in the middle third of the esophagus [1,3]. The development of EC is influenced by a complex interplay of factors, including smoking, alcohol consumption, dietary habits such as the intake of very hot foods and drinks, red meat, salted, or processed meats, obesity, genetic predispositions, and a family history of the disease [5–7]. Among these, the cumulative amount of alcohol consumption is identified as the primary risk factor [8,9], and there is evidence to suggest that specific types of alcohol, such as hard liquor, may pose a higher risk [10]. Despite recent advancements in the treatment and prevention of EC, the prognosis remains poor. The current staging system for EC by the American Joint Committee on Cancer (AJCC) and the International Union Against Cancer (UICC) is still the primary method for predicting prognosis [4,11].

PD-1 is an inhibitory receptor predominantly expressed on activated T cells, B cells, and certain natural killer (NK) cells. Its ligand, PD-L1, is primarily found on tumor cells and antigen-presenting cells (APCs). Under physiological conditions, the interaction between PD-1 and PD-L1 transmits inhibitory signals that suppress T cell activation. This mechanism serves to prevent excessive immune response expansion and protects the body from autoimmune diseases. During carcinogenesis, APCs utilize major histocompatibility complex (MHC) molecules to present tumor-specific antigens to the T cell receptor (TCR) on the surface of T cells. Following binding and specific recognition, effector T cells are ultimately activated through signaling pathways such as mitogen-activated protein kinase (MAPK), leading to the destruction of tumor cells [12].However, within the tumor microenvironment, when PD-1 on T cells interacts with PD-L1 on tumor cells or APCs, the immunoreceptor tyrosine-based inhibitory motifs (ITIMs) and immunoreceptor tyrosine-based switch motifs (ITSMs) of PD-1 become phosphorylated.This process recruits and activates SHP-2, which effectively inhibits T cell activation. Consequently, this can lead to T cell apoptosis, reduced cytokine production, impaired T cell lysis, and the induction of antigen tolerance. As a result, tumors are able to evade immune surveillance [13].Notably, recent numerous studies have shown that the PD-1/PD-L1 signaling pathway is intimately linked to tumorigenesis and progression, and blocking this pathway has emerged as a promising target for cancer immunotherapy [14–16]. However, there is a scarcity of research on PD-1/PD-L1 in EC, with existing studies presenting conflicting findings on the relationship between PD-1/PD-L1 expression and prognosis. This study aims to investigate the expression levels of immune cells related to the PD-1 pathway in EC to assess their prognostic significance and to inform clinical practice.

## 1. Materials and methods

### 1.1. Patients

In this study, we recruited a total of 236 individuals diagnosed with EC at our institution from January 1, 2016 to January 1, 2021. Inclusion criteria encompassed: (1) histopathologically confirmed EC; (2) initial diagnosis of the condition; (3) availability of comprehensive clinical records. Patients were excluded if they presented with: (1) indeterminate pathological types or clinical staging; (2) synchronous other malignancies; (3) psychiatric disorders; (4) pregnancy or lactation. The following data were meticulously collected: gender, age, body mass index (BMI), tumor size, TNM stage in accordance with the 8th edition of the UICC/AJCC staging system, lymph node metastasis, histological differentiation grade, tumor invasion depth and overall survival (OS) from follow-up [4]. The authors accessed pathological specimens from all patients between 01/02/2024 and 20/03/2024. The authors had access to information that could identify individual participants during or after data collection. The TNM staging system categorizes EC into various stages based on the depth of tumor invasion (T), the presence and number of regional lymph node metastases (N), and the presence of distant metastasis (M). Specifically, T1 to T4 indicates the increasing depth of tumor invasion from the mucosa to the adventitia of the esophagus or adjacent structure. N0 signifies no regional lymph node metastasis, while N1, N2, and N3 denote 1–2, 3–6, and seven or more regional lymph node metastases, respectively. M0 and M1 represent the absence or presence of distant metastasis. Patients were stratified into I-II stage and III-IV stage groups. Patients with I-II stage were characterized by T1-3N0M0 tumors or T1N1M0 tumors, indicating limited tumor invasion and no or minimal lymph node involvement without distant metastasis. Patients with III-IV stage included those with any T N2-3 M0 tumors, indicating extensive lymph node involvement, or T2-4 N1 M0 tumors, suggesting more profound tumor invasion with lymph node involvement but without distant spread. Additionally, any T any N M1 tumors were classified as advanced, indicating the presence of distant metastasis regardless of tumor invasion or lymph node status. The patient information is shown in S1 Table. This study was approved by the hospital’s ethics committee(Approval Number: KYLLLKS20230045 (March 17,2023), and all patients or their legal representatives provided written informed consent.

### 1.2. Immunohistochemistry

Immunohistochemical analysis was conducted using the streptavidin-perosidase method to assess the protein expression of PD-1, PD-L1, FOXP3, CD4, CD8 and CD25 in cancer tissues and adjacent tissues. The adjacent tissues of the tumor were utilized as the control group.After de-paraffinization, formalin-fixed, paraffin-embedded sections from the study participants were treated with 3% hydrogen peroxide for 10 minutes to quench endogenous peroxidase activity, followed by antigen retrieval through high-temperature and high-pressure for 15 minutes. After three rinses with phosphate buffer, sections were blocked with goat serum for 10 minutes at 37°C. The primary antibodies, including PD-1 (MAB-0734, Maixin), PD-L1 (22C3, M3653, Dako), FOXP3 (MAB-1004, Maixin), CD4 (RMA-0620, Maixin), CD8 (RMA-0514, Maixin) and CD25 (ZA-0590, Zhong Shan-Golden Bridge), were then applied and incubated overnight at 4°C, respectively. Following three more phosphate buffer washes, the sections were incubated with the secondary antibody, goat anti-rabbit antibody (Roche) or goat anti-mouse antibody (Roche), at 37°C for 15 minutes. After three phosphate buffer washes, excess fluid was removed, and sections were stained with 3,3’-diaminobenzidine (Roche). Then the sections were counterstained with hematoxylin, and dehydrated through a graded ethanol series, and air-dried before being sealed with neutral gum. The stained sections were analyzed using a microscope. Staining intensity was scored as follows: negative, 0; weak, 1; moderate, 2; strong, 3. The scoring criteria for the percentage of positive cells were: < 5%, 0; 5%−25%, 1; 26%−50%, 2; 51%−75%, 3; > 75%, 4. The sum of these two scores represented the overall positivity score for the case, with 0–2 indicating low expression and >2 indicating high expression.

### 1.3 Statistical analysis

Data analysis was performed using R4.0.3, with count data expressed as percentages (%). Spearman’s rank correlation analysis was used to evaluate the relationship between the expression levels of the six immunohistochemical markers and clinical pathological features. To assess the effect of immunohistochemical marker expression on patient survival, univariate analysis was conducted using the Kaplan-Meier method. This method estimates survival probabilities at various time points, creating a stepwise plot where each step down represents an event occurrence [17]. The LOG-rank test was then applied to compare survival curves between groups, providing a non-parametric statistical assessment of the differences in survival times. It calculates the expected number of events under the null hypothesis of equal survival probabilities across groups and compares these with the observed events to determine statistical significance [17]. Furthermore, the forward LR method was employed to select variables with statistical significance from the univariate analysis, which were then used to construct a clinical prediction model (multifactorial Cox model). The results of the multifactorial analysis and predictions for 1/2/3-year survival rates were graphically presented using bar charts. The predictive efficiency of the model was assessed using a calibration curve, receiver operating characteristic (ROC) curve, and the area under the ROC curve (AUC) for 1/2/3 years. A decision curve analysis (DCA) was plotted to evaluate the clinical utility of the prediction model. The significance level was set at α = 0.05 (two-tailed).

## 2. Results

### 2.1. Correlation between immunohistochemical markers and clinical features

**Fig 1:**
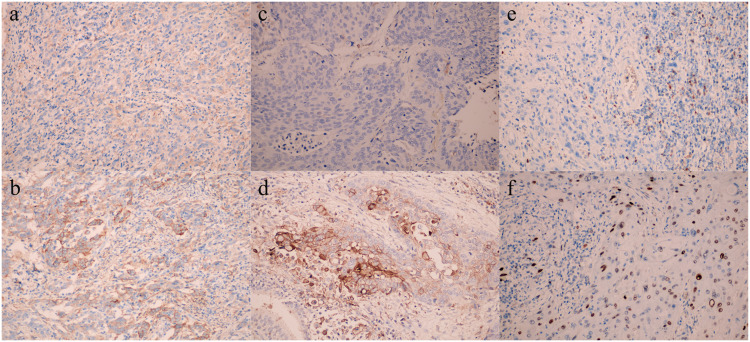
Comparison of protein expression of PD-1, PD-L1 and FOXP3 in esophageal cancer tissues ((a) PD-1 Low expression; (b) PD-1 High expression; (c) PD-L1 Low expression; (d) PD-L1 High expression; (e) FOXP3 Low expression; (f) FOXP3 High expression).

**Fig 2:**
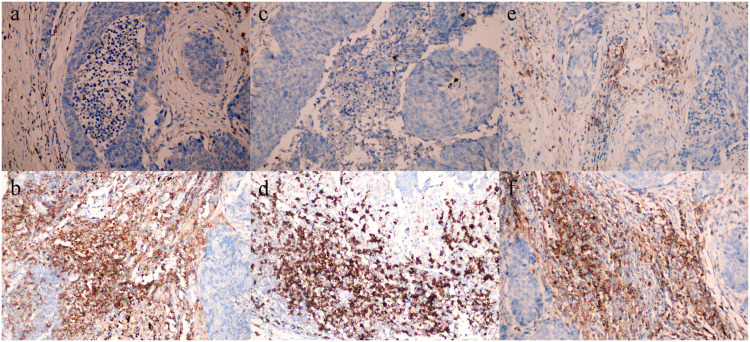
Comparison of Tregs infiltration in esophageal cancer tissues ((a) CD4 Low expression; (b) CD4 High expression; (c) CD8 Low expression; (d) CD8 High expression; (e) CD25 Low expression; (f) CD25 High expression).

In a cohort of 236 patients subjected to immunohistochemical analysis, high expression of PD-1, PD-L1, FOXP3, CD4, CD8, and CD25 were observed in 109, 106, 125, 127, 153, and 121 patients, respectively (S2 Table). In the adjacent tissues of the cancer, all variables exhibited low or negative expression, with the exception of PD-L1, which showed no expression.Representative immunohistochemical images illustrating the expressions of these markers are displayed in Figs 1 and 2, S1 Fig. Further analysis was conducted to explore the correlation between the expression of these immunohistochemical markers and clinical features (S3 Table.). Our findings revealed a significant positive association between the expression of PD-1, PD-L1, FOXP3, and CD25 with the depth of tumor invasion and the presence of lymph node metastasis (P < 0.05). In contrast, the expression levels of CD4 and CD8 exhibited a significant negative association with these clinical parameters (P < 0.05). Moreover, we observed an inverse relationship between the degree of tumor differentiation and the expression levels of PD-1, PD-L1, FOXP3, and CD25 (P < 0.05), while a direct relationship was noted for CD4 and CD8 expression levels (P < 0.05).

### 2.2. Analysis of prognostic factors in EC patients

To identify prognostic indicators associated with EC, we conducted univariate and multivariate analyses. The univariate analysis indicated that tumor diameter, degree of tumor differentiation, TNM stage, depth of tumor invasion, lymph node metastasis, and the expression levels of PD-1, PD-L1, FOXP3, CD25, CD4, and CD8 were factors significantly influencing EC prognosis (P < 0.05) (Table 1). Subsequently, a Cox multivariate regression analysis was performed, treating the aforementioned factors as independent variables and prognosis as the dependent variable. The results indicated that large tumor diameter, poor tumor differentiation, deep tumor invasion, lymph node metastasis, and high expression of PD-1, PD-L1, FOXP3, and CD25 were independent risk factors for EC prognosis (P < 0.05) (Table 1). Notably, high tumor differentiation and high expression levels of CD4 and CD8 were significantly correlated with a better prognosis for EC (P < 0.05) (S2 Fig.).

**Table 1:** Univariate and multivariate analysis of prognostic factors.

Variable	Univariate analysis			Multivariate analysis		
	Log-Rank test $X^{2}$	95.0% CI	P	HR	95.0% CI	P
Age	0.069		0.793	–	–	–
<60		27.691-31.544				
≥60		26.735-29.984				
Sex	0.223		0.637	–	–	–
Male		27.130-30.024				
Female		27.486-32.014				
BMI(kg/m^2^)	1.058		0.304	–	–	–
<24		25.581-29.784				
≥24		28.038-31.110				
Blood type	1.209		0.751	–	–	–
A		24.627-30.444				
B		27.844-31.537				
O		26.121-31.362				
AB		25.865-31.721				
Smoke history	0.246		0.620	–	–	–
Yes		27.981-32.751				
No		27.034-29.893				
History of alcohol	0.165		0.685	–	–	–
Yes		27.307-30.273				
No		26.513-31.126				
Family history of cancer	1.765		0.184	–	–	–
Yes		25.225-34.108				
No		27.408-30.011				
Tumor type	0.656		0.720	–	–	–
Squamous		27.363-30.050				
Adenocarcinoma		22.281-33.173				
Adenosquamous cell carcinoma		27.151-34.849				
Tumor location	3.657		0.161	–	–	–
Upper		19.315-29.230				
Middle		27.245-30.417				
Lower		28.070-32.249				
Number of tumors	0.920		0.631	–	–	–
1		27.334-30.587				
2		26.236-30.673				
3		24.384-33.616				
Esophageal fistula	0.761		0.383	–	–	–
Yes		28.389-32.229				
No		26.815-29.852				
Nerve invasion	0.229		0.632	–	–	–
Yes		25.940-31.030				
No		27.483-30.356				
Vessel invasion	0.365		0.546	–	–	–
Yes		24.991-30.665				
No		27.734-30.499				
Chemotherapy sensitivity	0.577		0.447	–	–	–
Sensitive		26.989-30.001				
Insensitivity		27.345-31.843				
Tumor diameter(mm)	6.943		0.008	2.161	1.430-3.266	< 0.001
<5		29.721-32.732				
≥5		24.924-28.639				
Degree of tumor differentiation	7.877		0.019			
Low		18.460-27.326				
Median		25.774-30.272		0.544	0.2994-0.990	0.046
High		28.989-31.943		0.308	0.1421-0.668	0.002
Depth of tumor invasion	9.63		0.002	1.805	1.0348-3.149	0.037
T1 + T2		31.149-34.266				
T3 + T4		25.721-28.866				
Lymphnode metastases	4.754		0.029	3.967	2.0066-7.843	< 0.001
Metastases		24.846-29.612				
None		28.195-31.060				
PD-1	8.342		0.004	2.750	1.4104-5.364	0.003
Low		29.722-32.580				
High		24.040-28.065				
PD-L1	7.427		0.006	2.974	1.4792-5.980	0.002
Low		29.567-32.386				
High		24.036-28.209				
FOXP3	8.757		0.003	2.400	1.1790-4.886	0.015
Low		30.481-33.246				
High		24.192-27.965				
CD4	5.064		0.024	0.195	0.0997-0.382	< 0.001
Low		25.714-29.531				
High		28.162-31.448				
CD8	3.936		0.047	0.272	0.1304-0.568	< 0.001
Low		25.559-29.807				
High		27.849-30.947				
CD25	6.994		0.008	1.697	1.0286-2.801	0.038
Low		29.008-32.377				
High		25.200-28.793				

### 2.3. Survival analysis

**Fig 3:**
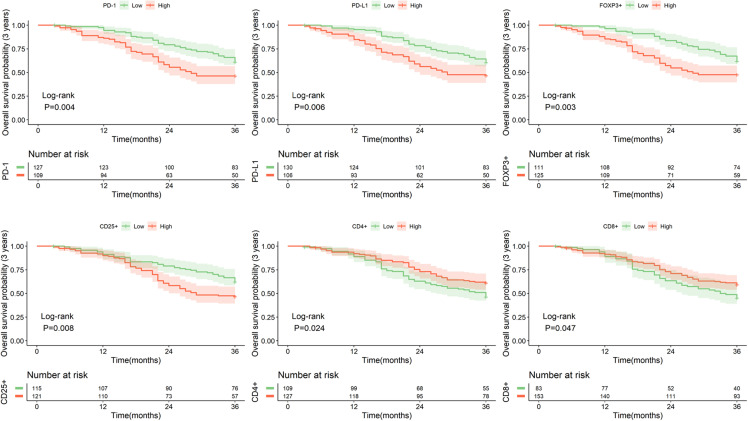
Effect of immunohistochemical indicator expression levels on patient survival rates.

Then the Kaplan-Meier and LOG-rank test were used to analyze the impact of the expression levels of the six immunohistochemical markers on the three-year survival rate of patients. The findings revealed that patients with lower expression levels of PD-1, PD-L1, FOXP3, CD25, and higher expression levels of CD4 and CD8 had improved three-year survival rates, with statistically significant differences (P < 0.05) (Fig 3). Furthermore, in light of previous studies highlighting the dichotomous influence of PD-L1 on patient prognosis [18,19], we conducted analysis of the prognostic impact of PD-L1 expression across different stages of EC. As depicted in S3 Fig, we observed that in stage I-II disease, patients with higher PD-L1 expression have better OS compared to those with lower PD-L1 expression (P < 0.05). However, in stage III-IV disease, patients with higher PD-L1 expression fared worse in terms of OS than those with lower PD-L1 expression (P < 0.05).

### 2.4. Nomogram construction

**Fig 4:**
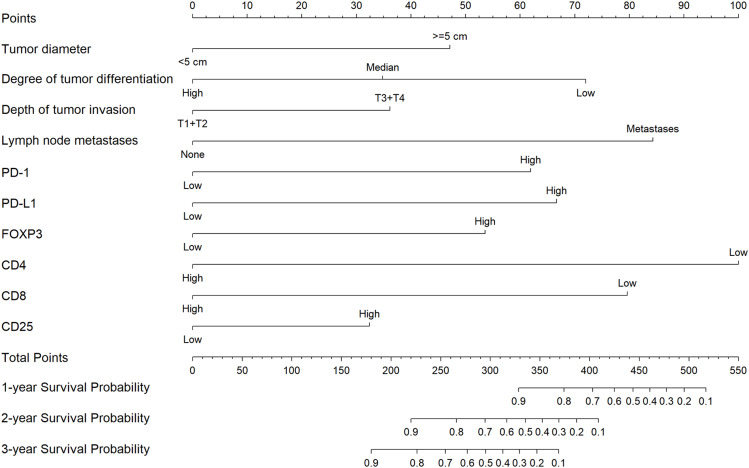
Nomogram for predicting 1-year, 2-year, and 3-year survival rates of esophageal cancer.

Based on the factors selected by the multivariable Cox regression analysis, including tumor diameter, tumor differentiation degree, tumor invasion depth, lymph node metastasis, PD-1, PD-L1, FOXP3, CD25, CD4, and CD8 as independent variables, a nomogram was constructed (Fig 4). Each variable corresponds to a point scale, and based on the total score, one-year, two-year, and three-year survival rates for EC patients can be determined.

### 2.5. Validation of the prediction model

**Fig 5:**
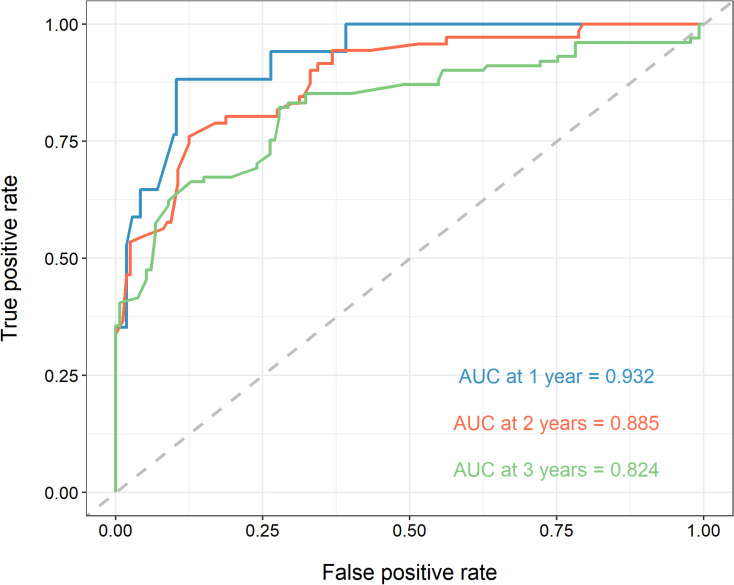
ROC curve demonstrated that the area under the curve for the one-year, two-year, and three-year survival rates.

**Fig 6:**
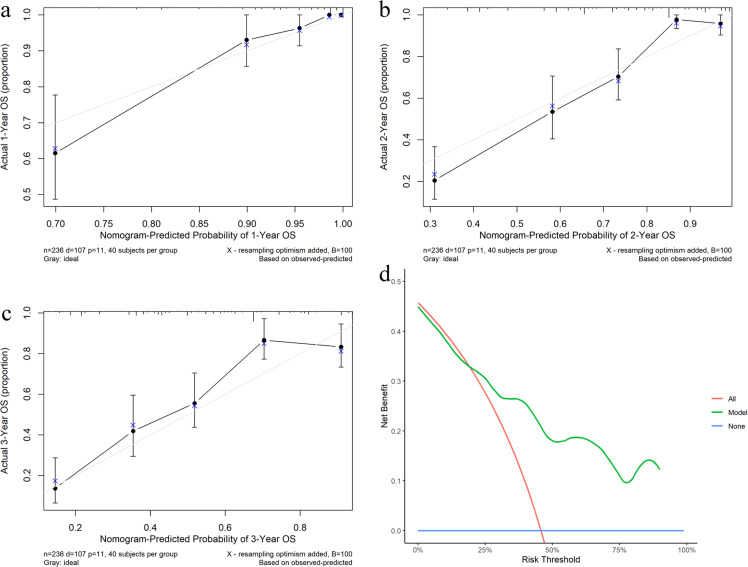
The calibration curve predicting 1- (a), 2- (b), and 3- year (c) survival in patients with esophageal cancer; (d) decision curve analysis.

According to the results of the Cox regression analysis, a predictive model for the OS rate was developed using the aforementioned factors. The ROC curve (Fig 5) demonstrated that the area under the curve for the one-year, two-year, and three-year survival rates were 0.932, 0.885, and 0.824, respectively. The predictive sensitivities for the one-year, two-year, and three-year survival rates were 0.882, 0.760, and 0.8217, with specificities of 0.896, 0.875, and 0.7218, indicating the model’s robust predictive performance. The calibration curve showed that the survival rates predicted by the nomogram closely aligned with the actual one-year, two-year, and three-year survival rates, demonstrating good concordance. The DCA revealed that the clinical utility of this research model was superior to the no-intervention group under various threshold probability conditions, except for a slight decrease in the 0 to 0.20 threshold probability range. This suggests that using the nomogram developed in this study to predict the survival rate of EC patients would result in significant net benefits and holds substantial clinical relevance (Fig 6).

## 3. Discussion

Immunotherapy has emerged as an innovative approach for the treatment of malignant tumors in recent years. It primarily aims to control and eliminate tumor cells by activating the body’s anti-tumor immune response, offering broad prospects for the treatment of malignant tumors. However, only a small subset of patients can benefit from this novel therapy [14,20–22]. The binding of PD-1 and PD-L1 can generate inhibitory signals, and some signaling pathways can induce tumor immune tolerance by inhibiting the activation of T cells [23,24]. Therefore, blocking these signaling pathways can lead to T cell activation and the destruction of tumor cells. Studies have demonstrated that elevated PD-1 and PD-L1 expression in tumor tissues is associated with reduced immune cell activity [15,25,26], which correlates with poor outcomes in cancers such as lung cancer [27], pancreatic cancer [28,29], and bladder cancer [30]. In EC, our findings align with those of Ohigashi et al. [26] and Chen et al. [31], showing that patients with high PD-L1 expression have a poorer prognosis than those with low expression. It is well known that PD-L1 can be detected in tumor cells (TC) and tumor-infiltrating immune cells (TIIC); however, their clinical significance differs. Kento Kawasaki et al. [32] and Hu J. et al. [33], employed multiplex immunohistochemistry to evaluate the expression of PD-L1 in both TC and lymphocytes. Their findings indicate that only the expression of PD-L1 in TC is associated with poor clinical outcomes, while the infiltration of PD-L1 + TIIC does not correlate with survival rates.Although our study did not perform multiple immunohistochemical analyses on the expression of PD-L1 in TC and TIIC, unlike the two aforementioned studies,we also observed that in I-II stage, patients with high PD-L1 expression exhibited better OS than those with low PD-L1 expression, which is in line with previous research [18,19]. However, in III-IV stage, we found that patients with high PD-L1 expression had poorer OS than those with low PD-L1 expression. The mechanism by which PD-L1 expression affects clinical outcomes in EC patients remains unclear. It is plausible to hypothesize that PD-L1 expression may represent the presence of an antitumor response.Studies [12] have demonstrated that the elevated expression of PD-L1 on tumor cells can interact with PD-1 present on the surface of T cells, thereby inhibiting T cell activation and proliferation. This interaction enables tumor cells to evade immune surveillance by the host. Phase III randomized controlled clinical trials [34–36] and meta-analyses [37] focused on this pathway have confirmed that blocking the binding between PD-L1 and PD-1 using immune checkpoint inhibitors restores T cells’ ability to recognize and eliminate tumor cells. Consequently, this enhances anti-tumor immune responses, ultimately resulting in significant improvements in objective response rates, progression-free survival, and overall survival among patients. In our study, the positive expression of PD-L1 exhibited inconsistent prognostic implications in patients with EC across various stages.We hypothesize that in the early stages of cancer, tumor growth might still be partially suppressed, leading to better survival. However, in the later stages, the resistance mechanisms may ultimately become ineffective, permitting tumor cells to survive and progress. Nevertheless, the specific underlying mechanisms warrant further investigation.It is also important to note that Guo et al.’s study demonstrated that patients with PD-L1 positive tumors had better OS compared to those with PD-L1 negative tumors [18]. These divergent results may be attributed to differences in antibody sensitivity, PD-L1 staining positivity cut-off values, and heterogeneity in tumor PD-L1 expression affecting staining results [38]. Furthermore, we observed a close relationship between the expression levels of PD-1 and PD-L1 and tumor infiltration depth and lymph node metastasis. Additionally, neurological invasion, vascular invasion, and tumor infiltration depth have been confirmed by many researchers as independent risk factors for the prognosis of EC patients [39,40], a conclusion that is also supported by our study. These results suggest that PD-1 and PD-L1 may play a role in EC progression and serve as significant prognostic markers.

Tumor lymphocyte infiltration is considered a manifestation of the body’s anti-tumor immune response, and tumor-infiltrating cells play important roles in the occurrence and development of tumors and can predict the prognosis of a variety of tumors. As is well known, the role of CD4 + T cells in generating potent CD8 + effector cells has long been established [41,42]. In some preclinical [41,43] and clinical studies [43], the anti-tumor function of CD4 + T cells in the absence of CD8 + T cells has been reported, indicating a more direct role of CD4 + T cells in anti-tumor immunity. During the effector phase of anti-tumor immunity, CD4 + lymphocytes can directly kill tumor cells while releasing cytokines to activate tumor-killing immune cells, indirectly eliminating tumor cells. Therefore, CD4 + T lymphocytes demonstrate a positive anti-tumor effect in EC, and elevated levels of CD4 + expression may be a good prognostic marker. CD8 + T cells are the terminal effector cells in cancer immunity that, through a series of fine regulations, cell proliferation, and differentiation, ultimately form cytotoxic T lymphocytes (CTLs). These CTLs can express perforins and granzymes, penetrate, and destroy virus-infected or tumor cell membranes. CD8 + T cells proliferate significantly in the tumor microenvironment, directly acting on tumor cells, inducing tumor cell apoptosis, and killing antigen-bearing target cells [44]. Numerous studies [45,46] have found that patients with lower CD4 + CD8 + cell ratios in EC tissues have lower overall survival compared to those with higher CD4 + CD8 + cell ratios. Our research results demonstrate that patients with high expression of CD4 and CD8 have better survival benefits, consistent with the above research findings. However, it is crucial to note the heterogeneity of CD4 + T cells, which encompasses various subsets, including helper T cells (such as Th1, Th2, Th17, etc.) that primarily activate and modulate the responses of other immune cells, and regulatory T cells (Treg), which play a suppressive role in tumor immunity [47].

Tregs are a subset of T cells expressing FOXP3, CD25, and CD4 as cell phenotypic characteristics, and play an important role in forming moderate immune responses and maintaining normal physiological functions in the body. Tregs accumulate in the peripheral circulation and tumor sites of patients, helping tumors evade the host immune system. This type of T cell shows a significant increase in the ratio in the peripheral blood of patients with various malignant tumors, including lung cancer [48] and breast cancer [49]. Studies [50] have suggested that compared to Tregs in peripheral blood, tumor-infiltrating Tregs exhibit more proliferative and immunosuppressive phenotypes, with increased expression of CTLA-4, CD25, GITR, 4−1BB, OX40, ICOS, LAG-3, TIM3, TIGIT, and PD-1. The presence of tumor-infiltrating regulatory T cells is associated with poor outcomes in solid cancers. FOXP3 is an important transcription factor in the development of Tregs. Research [51,52] has confirmed that FOXP3 plays an important role in the production, activation, and functional regulation of Tregs, while Tregs act as the executors of immune function, inhibiting the proliferation of CD8 + infiltrating T cells, disrupting the balance between T lymphocyte subgroups, suppressing immune function, and promoting immune escape. Research has found that FOXP3, although as a marker for T cells, is rarely expressed in healthy cell tissues but is aberrantly upregulated in various malignant tumors, such as lung cancer [53], pancreatic cancer [54], thyroid cancer [55], and T-cell lymphoma [56]. This upregulation is associated with a poor biological prognosis and is believed to facilitate tumor progression through multiple mechanisms. Conversely, some views suggest that high expression of the FOXP3 protein in breast cancer [51] and hepatocellular carcinoma [57] exerts tumor-suppressive effects. Our research results indicate that in EC, an elevated level of FOXP3 expression predicts a relatively poorer prognosis for patients, consistent with the conclusions of Wu et al. [58], suggesting that blocking FOXP3 expression in cancer cells may provide a new treatment strategy for EC. However, it is important to note that our study did not differentiate between FOXP3 staining in tumor cells and infiltrating immune cells. Given that FOXP3 is a marker for Tregs, and considering the limited research on FOXP3 expression in EC tumor cells, the relationship between FOXP3 expression in these cells and patient prognosis warrants further investigation.

CD25 is highly expressed in hematologic malignancies, activated circulating immune cells, and Tregs, while it is low in most solid tumors. However, Tregs typically infiltrate into solid tumors. Immunological studies [59,60] have shown that CD4 + CD25 + Tregs can suppress the immune response of T cells to foreign and self-antigens, thereby maintaining immune tolerance and inhibiting the immune response to tumor cells, ultimately leading to immune escape of tumor cells. Clinical studies have also confirmed that CD4 + CD25 + Tregs are recruited to tumor sites by recombinant human C-C motif chemokine 22 protein (CCL22) secreted by ovarian cancer cells, resulting in the accumulation of CD4 + CD25 + Tregs in cancer tissues. An excessive amount of CD4 + CD25 + Tregs can lead to immune escape and promote tumor development [61]. Our research found that high expression of CD25 is one of the adverse prognostic factors for EC patients. After adjusting for confounding factors, high expression of CD25 is positively correlated with the risk of patient death. This conclusion is consistent with the aforementioned animal experiments and clinical research results. This suggests that for cancer treatment, targeted anti-tumor drugs based on CD25 are expected to bring survival benefits to more EC patients. However, it is crucial to consider the complex role of CD25. CD25 is the alpha chain of the trimeric receptor of IL-2, and lack of CD25 not only causes autoimmunity due to immunosuppression but also increases the risk of many infections as IL-2 has pivotal roles in inducing Th1 and CD8 + cytotoxic T cells [62]. This feature is somewhat similar to CD8 T lymphocytes, in that it has been well-established for many years that not all CD8 + cells are cytotoxic and that there are subgroups with immunosuppressive activity [63].

Given the complexity of immunity, a better understanding of the tumor microenvironment and the use of additional biomarkers are necessary for improved prognosis prediction. We constructed a multifactorial prognostic model to predict 1-year, 2-year, and 3-year survival rates in EC, which showed strong predictive power upon validation. However, this study has limitations. Firstly, this study is a retrospective analysis and based on single-center data, which may introduce some bias and errors. Secondly, the absence of multiplex staining in our study precludes the precise co-localization of immune markers within the tumor microenvironment. Advanced staining techniques could have offered a more detailed understanding of the spatial relationships between different cell types and their potential interactions. Furthermore, we did not differentiate the expression of FOXP3 between tumor cells and immune cells. The distinct roles of FOXP3 in these cell populations could have significant implications for tumor biology and immune responses. Future studies with larger samples, advanced detection techniques, and prospective designs are needed to further explore the relationship between immune markers and EC.

In conclusion, there is a significant correlation between PD-1 pathway-related immune cells and clinical pathological features of EC; tumor size, poor tumor differentiation, deep tumor infiltration, lymph node metastasis, high expression of PD-1, PD-L1, FOXP3, and CD25 are independent risk factors affecting patient prognosis; and patients with high expression of CD4 and CD8 have better prognosis. A predictive model based on the expression levels of PD-1, PD-L1, FOXP3 proteins, and Tregs infiltration has high predictive effectiveness for the prognosis of EC patients, which may provide clues for exploring new treatment modalities for EC in the future.

## Supporting information

S1 Table Patient demographics and clinicopathologic factors (n = 236). (DOCX)

S2 Table The expression of immune-related indicators determined by immunohistochemistry assays. (DOCX)

S3 Table The relationship between immunohistochemical indicators and clinicopathological features. (DOCX)

S1 Fig Expression of six immune variables in adjacent tissues of cancer ((a) PD-1 Low expression; (b) PD-L1 Negative expression; (c) FOXP3 Low expression; (d) CD4 Low expression; (e) CD8 Low expression; (f) CD25 Low expression).
(TIF)

S2 Fig Forest plot of analysis of prognostic factors in esophageal cancer patients. (TIF)

S3 Fig Analysis of the prognostic impact of PD-L1 expression across different stages of esophageal cancer. (TIF)
