# Supplementary material for: The distribution characteristics of PD-1 pathway-related immune cells in esophageal cancer tissue and their prognostic significance
Source: PLoS One. 2025 Jun 30;20(6):e0325349. doi: 10.1371/journal.pone.0325349 (PMC12208471; doi:10.1371/journal.pone.0325349)
Supplement: S1 Table — (DOCX) [file pone.0325349.s001.docx]

**S1 Table. Patient demographics and clinicopathologic factors (n= 236).**

| Parameters | Number (n= 236) |
| --- | --- |
| Age (N, %) |  |
| ＜60 | 83 (35.17) |
| ≥60 | 153 (64.83) |
| Sex (N, %) |  |
| Male | 192 (81.36) |
| Female | 44 (18.64) |
| BMI（kg/m^2^） (N, %) |  |
| ＜24 | 97 (41.10) |
| ≥24 | 139 (58.90) |
| Blood type (N, %) |  |
| A | 57 (24.15) |
| B | 84 (35.59) |
| O | 66 (27.97) |
| AB | 29 (12.29) |
| Smoke history (N, %) |  |
| Yes | 194 (82.20) |
| No | 42 (17.80) |
| History of alcohol (N, %) |  |
| Yes | 175 (74.15) |
| No | 61 (25.85) |
| Family history of cancer (N, %) |  |
| Yes | 22 (9.32) |
| No | 214 (90.68) |
| Tumor type (N, %) |  |
| Squamous | 211 (89.41) |
| Adenocarcinoma | 11 (4.66) |
| Adenosquamous cell carcinoma | 14 (5.93) |
| Tumor location (N, %) |  |
| Upper | 22 (9.32) |
| Middle | 143 (60.59) |
| Lower | 71 (30.09) |
| Number of tumors (N, %) |  |
| 1 | 139 (58.90) |
| 2 | 78 (33.05) |
| 3 | 19 (8.05) |
| Esophageal fistula (N, %) |  |
| Yes | 55 (23.31) |
| No | 181 (76.69) |
| Nerve invasion (N, %) |  |
| Yes | 67 (28.39) |
| No | 169 (71.61) |
| Vessel invasion (N, %) |  |
| Yes | 59 (25.00) |
| No | 177 (75.00) |
| Chemotherapy sensitivity (N, %) |  |
| Sensitive | 172 (72.88) |
| Insensitivity | 64 (27.12) |
| Tumor diameter（mm） (N, %) |  |
| ＜5 | 108 (45.76) |
| ≥5 | 128 (54.24) |
| Degree of tumor differentiation (N, %) |  |
| Low | 28 (11.86) |
| Median | 74 (31.36) |
| High | 134 (56.78) |
| Depth of tumor invasion (N, %) |  |
| T1+T2 | 170 (72.03) |
| T3+T4 | 66 (27.97) |
| Lymphnode metastases (N, %) |  |
| Metastases | 83 (35.17) |
| None | 153 (64.83) |
| TNM stage |  |
| I+II | 139 (58.90) |
| III+IV | 97(41.10) |
