# Supplementary material for: The distribution characteristics of PD-1 pathway-related immune cells in esophageal cancer tissue and their prognostic significance
Source: PLoS One. 2025 Jun 30;20(6):e0325349. doi: 10.1371/journal.pone.0325349 (PMC12208471; doi:10.1371/journal.pone.0325349)
Supplement: S2 Table — (DOCX) [file pone.0325349.s002.docx]

**S2 Table. The expression of immune-related indicators determined by immunohistochemistry assays.**

| Indicators | Number (n= 236) |
| --- | --- |
| PD-1 (N, %) |  |
| Low | 127 (53.81) |
| High | 109 (46.19) |
| PD-L1 (N, %) |  |
| Low | 130 (55.08) |
| High | 106 (44.92) |
| FOXP3 (N, %) |  |
| Low | 111 (47.03) |
| High | 125 (52.97) |
| CD4 (N, %) |  |
| Low | 109 (46.19) |
| High | 127 (53.81) |
| CD8 (N, %) |  |
| Low | 83 (35.17) |
| High | 153 (64.83) |
| CD25 (N, %) |  |
| Low | 115 (48.73) |
| High | 121 (51.27) |
