# Supplementary material for: The distribution characteristics of PD-1 pathway-related immune cells in esophageal cancer tissue and their prognostic significance
Source: PLoS One. 2025 Jun 30;20(6):e0325349. doi: 10.1371/journal.pone.0325349 (PMC12208471; doi:10.1371/journal.pone.0325349)
Supplement: S3 Table — (DOCX) [file pone.0325349.s003.docx]

**S3 Table.The relationship between immunohistochemical indicators and clinicopathological features.**

| Variable | PD-1 | PD-L1 | FOXP3 | CD4 | CD8 | CD25 |
| --- | --- | --- | --- | --- | --- | --- |
| PD-1 | 1 | 0.506** | 0.582** | -0.117 | -0.153* | 0.519** |
| PD-L1 | 0.506** | 1 | 0.509** | -0.013 | -0.124 | 0.437** |
| FOXP3 | 0.582** | 0.509** | 1 | -0.085 | -0.091 | 0.536** |
| CD4 | -0.117 | -0.013 | -0.085 | 1 | 0.469** | -0.072 |
| CD8 | -0.153* | -0.124 | -0.091 | 0.469** | 1 | -0.186** |
| CD25 | 0.519** | 0.437** | 0.536** | -0.072 | -0.186** | 1 |
| Depth of tumor invasion | 0.221** | 0.256** | 0.283** | -0.025** | -0.057** | 0.222** |
| Lymphnode metastases | 0.174** | 0.158* | 0.190** | -0.243** | -0.259** | 0.195** |
| Nerve invasion | 0.118 | 0.003 | 0.105 | -0.150 | -0.152 | -0.029 |
| Vessel invasion | 0.166 | 0.046 | 0.163 | -0.111 | -0.1 | 0.022 |
| Tumor diameter | 0.139* | 0.046 | 0.117 | -0.052 | 0.061 | 0.115 |
| Degree of tumor differentiation | -0.378** | -0.363** | -0.295** | 0.167* | 0.327** | -0.307** |

**＜0.01（bilateral）, significant correlation.

*＜0.05（bilateral）, significant correlation.
