# Supplementary figures and images for: The distribution characteristics of PD-1 pathway-related immune cells in esophageal cancer tissue and their prognostic significance
Source: PLoS One. 2025 Jun 30;20(6):e0325349. doi: 10.1371/journal.pone.0325349 (PMC12208471; doi:10.1371/journal.pone.0325349)

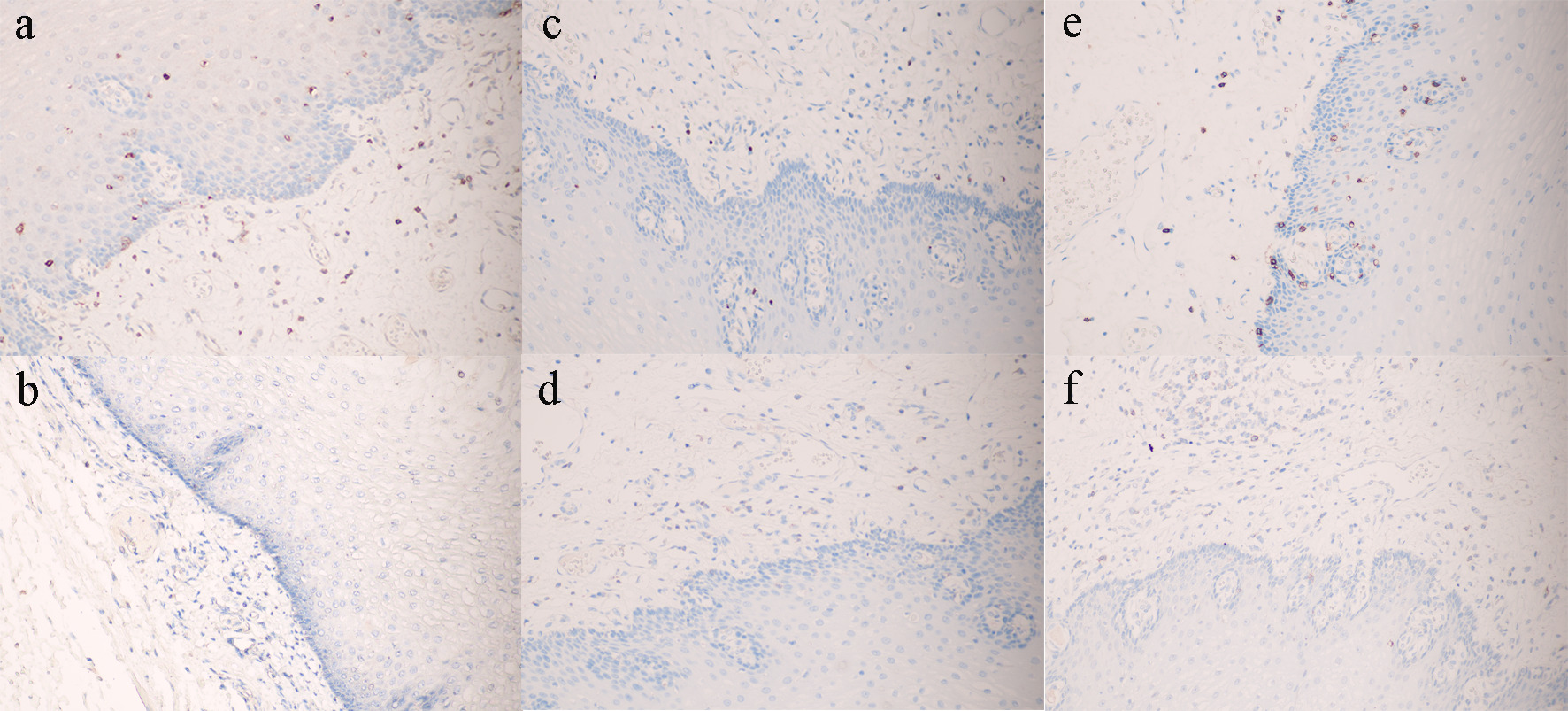

Supplement: S1 Fig — ((a) PD-1 Low expression; (b) PD-L1 Negative expression; (c) FOXP3 Low expression; (d) CD4 Low expression; (e) CD8 Low expression; (f) CD25 Low expression). (TIF) [file pone.0325349.s004.tif]

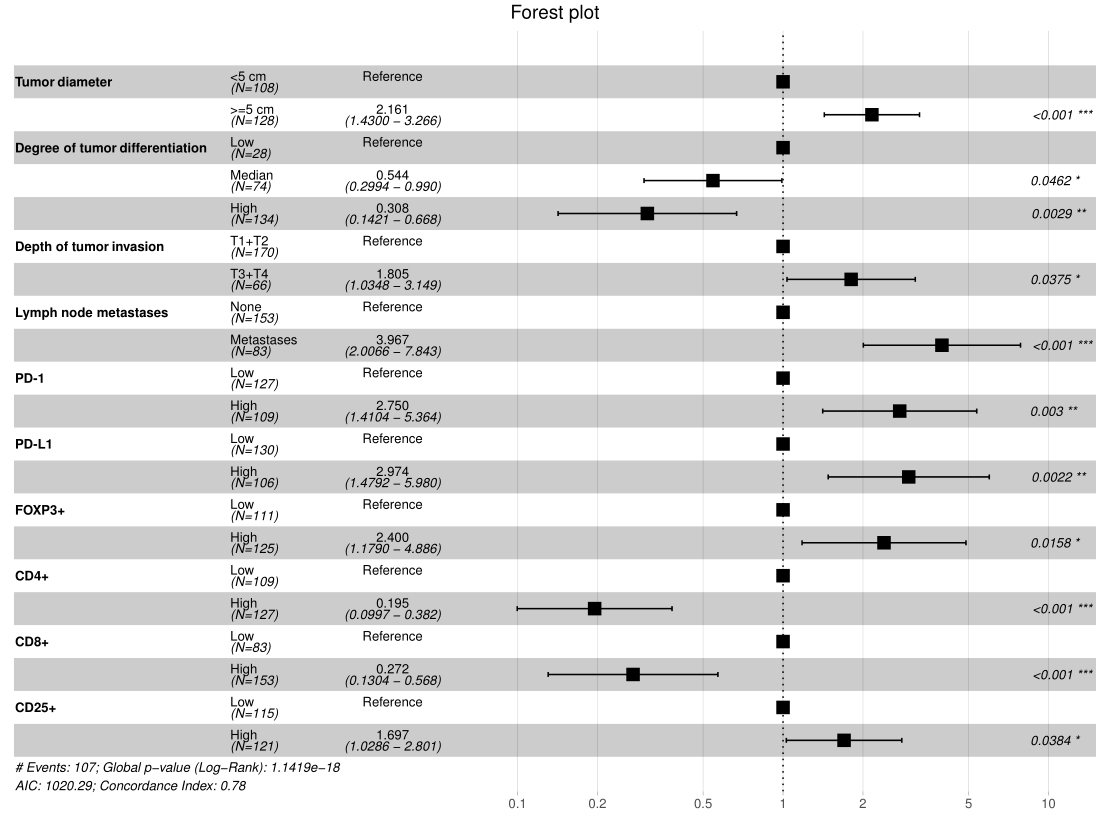

Supplement: S2 Fig — (TIF) [file pone.0325349.s005.tif]

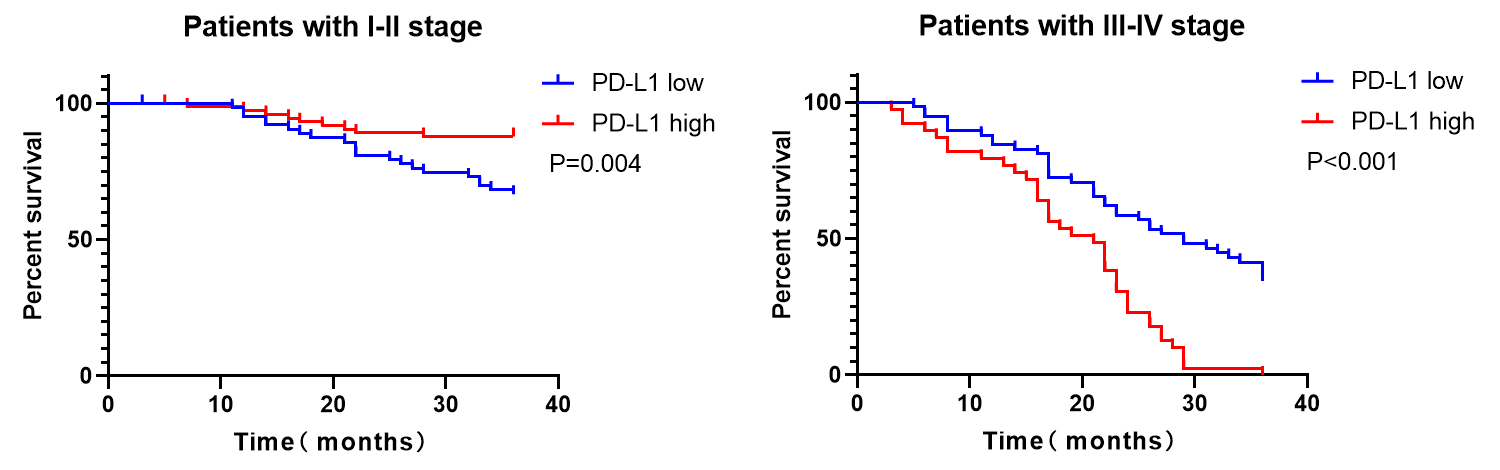

Supplement: S3 Fig — (TIF) [file pone.0325349.s006.tif]
